# Non-invasive assessment of interstitial myocardial fibrosis in pressure-overload left ventricular hypertrophy

**DOI:** 10.1186/1532-429X-14-S1-O5

**Published:** 2012-02-01

**Authors:** Andrew Jabbour, Tevfik F Ismail, Sofia V De Noronha, Carl Shakespeare, Sameer Zaman, Oluwatosin Sotubo, Saman S Zaman, Callum Ettles, Benjamin Hewins, Rick Wage, Ankur Gulati, Pedro F Ferreira, Pierre Croisille, Yanqiu Feng, Katsuya Norita, Raad H Mohiaddin, Taigang He, John Pepper, David N Firmin, Mary Sheppard, Dudley J Pennell, Mario Petrou, Sanjay K Prasad

**Affiliations:** 1 NIHR Cardiovascular Biomedical Research Unit, Royal Brompton and Harefield NHS Foundation Trust, Imperial College London, London, UK; 2 INSERM, Lyon, France; 3 Cardiothoracic Surgery, Royal Brompton Hospital and Imperial College London, London, UK; 4 Pathology Department, Royal Brompton Hospital and Imperial College London, London, UK

## Background

Aortic stenosis (AS) and systemic hypertension (HT) are associated with increased interstitial myocardial fibrosis (IMF). T1-mapping-derived extracellular volume fractions (Ve) have been shown to correlate with IMF in AS patients after infusion of gadolinium. We hypothesized that interstitial expansion could be detected after bolus gadolinium administration in patients with HT or severe AS when compared to healthy controls; and that these measures would correlate with both histological assessment of fibrosis from LV biopsies and abnormal myocardial strain.

## Methods

A Modified Look-Locker Inversion Recovery (MOLLI) sequence was used to generate 11 T1-weighted images in 3 groups of subjects: 1) Healthy Volunteers , 2) Severe AS, 3)Patients with significant but controlled hypertension. Myocardial and blood pool T1 values were derived by fitting a signal intensity-time curve using CMR42®. The λ was determined by plotting (1/T1myo vs. 1/T1blood pool) at various time points once contrast equilibrium was reached. Ve was derived accounting for hematocrit. Multiple short- and long-axis T1 maps were acquired at 1.5T (Siemens, Erlangen, Germany) before and 1,2,5,8,15,20,25 and 30 minutes after contrast. Myocardial tagging was acquired using single- and multiple-breath-hold CSPAMM sequences in multiple planes and analyzed with inTag® (Lyon, France). Histological validation was performed by biopsy of the LV septal and free walls during AV replacement surgery.

## Results

**Figure 1:**
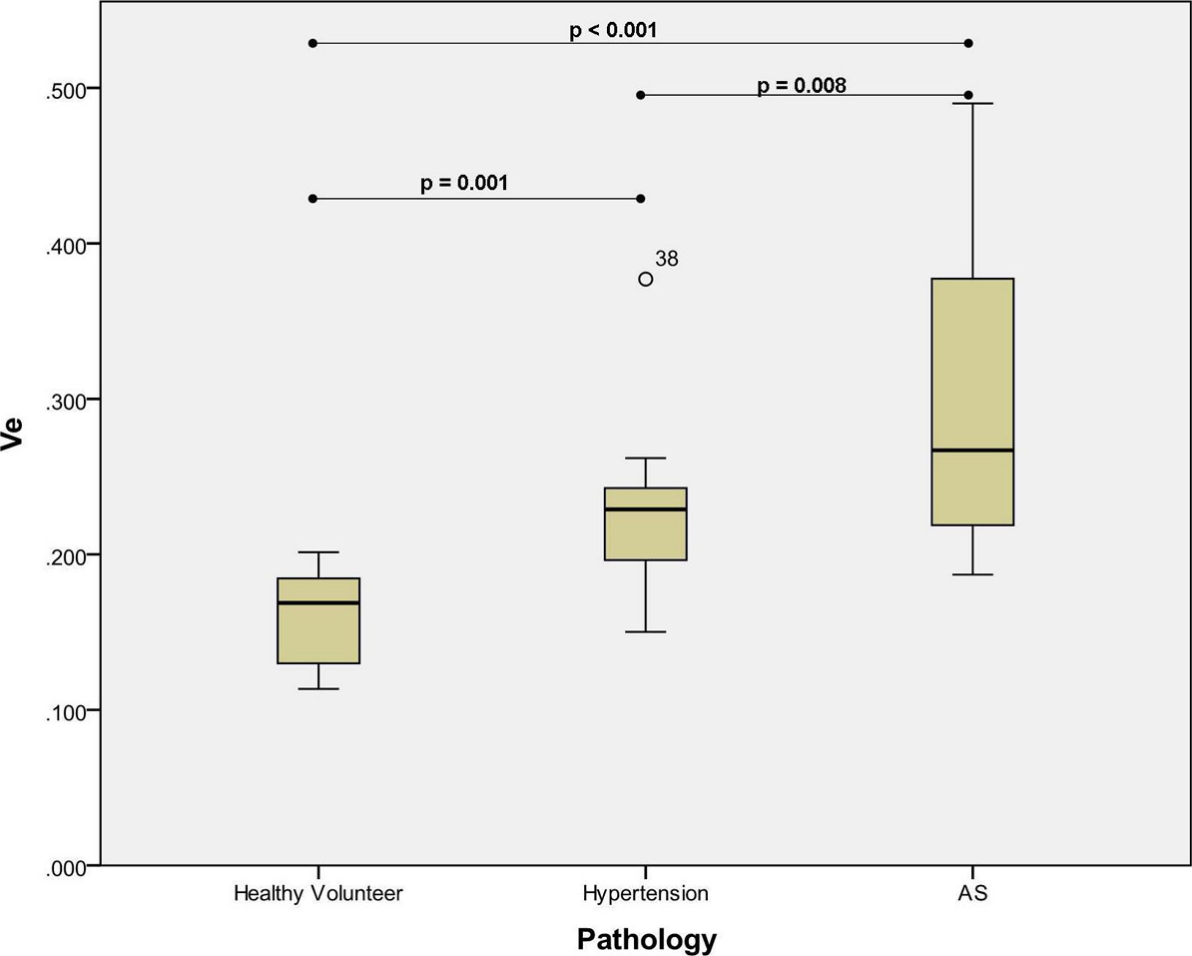


**Figure 2:**
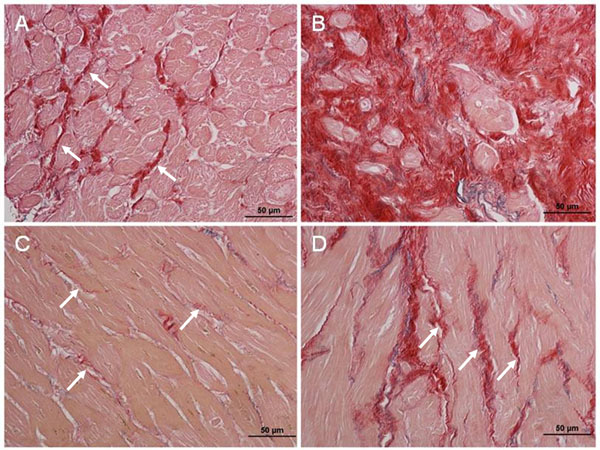


Sixty-three subjects (Severe AS n= 25,Hypertension n=19, Healthy control n=19) were recruited to the study. Subjects with HT and severe AS displayed higher Ve when compared to healthy controls (p<0.01), with AS patients demonstrating higher Ve than those with HT (p=0.008, Figure 1). AS patients with elevated Ve also displayed increased levels of both interstitial and replacement fibrosis on histology (Figures 2A & 2B, respectively). Significant heterogeneity in fibrosis burden existed with LV anterior free wall fibrosis (Fig. 2C) 5x lower when compared with the septum (Fig 2D). The Ve correlated with indices of reduced myocardial function including reduced circumferential δ strain (r=-0.69, p=0.001), angle peak strain (r=-0.48, p=0.04) and radial δ strain (r=-0.59, p=0.03); and increased left atrial dilatation (r=0.64, p=0.001). Inter- and intraobserver coefficients of variation were 3.2% and 5.0% respectively.

## Conclusions

Multi-slice T1-mapping-derived Ve is significantly elevated in patients with AS and also in those with systemic hypertension compared to healthy controls and correlates well with histology and indices of reduced myocardial performance. Multi-slice T1-mapping Ve measurement after bolus gadolinium administration is clinically practical and holds promise for the detection of IMF in pressure-overload hypertrophy.

## Funding

This project was supported by the NIHR Cardiovascular Biomedical Research Unit of Royal Brompton and Harefield NHS Foundation Trust, the British Heart Foundation, and CORDA. Dr. Jabbour was supported by a Postdoctoral.

Research Fellowship from the National Health and Medical Research Council of Australia, a Vincent Fairfax Family Foundation Research Fellowship from the Royal Australasian College of Physicians, the St. Vincent’s Clinic Foundation, and the Victor Chang Cardiac Research Institute.

